# Point-of-Care Ultrasonography and Worsening of Renal Function in Acute Heart Failure: A Cohort Study

**DOI:** 10.1177/20543581251328069

**Published:** 2025-07-29

**Authors:** Guillaume Soret, Antonio Leidi, Alexandre Leszek, Christophe Marti, Sebastian Carballo, Jérôme Stirnemann, Olivier Grosgurin, Jean-Luc Reny, Thomas A. Mavrakanas

**Affiliations:** 1 General Internal Medicine, Department of Medicine, Geneva University Hospitals, Switzerland; 2 Department of Internal Medicine, GHOL-Nyon Hospital, Switzerland; 3 Emergency Medicine, Department of Acute Medicine, Geneva University Hospitals, Switzerland; 4 Division of Nephrology and Experimental Medicine, Department of Medicine, McGill University Health Centre, Montreal, QC, Canada

**Keywords:** point-of-care ultrasonography, POCUS, acute heart failure, acute kidney failure, lung congestion

## Abstract

**Introduction:**

The goal of this study was to investigate the association between worsening renal function (WRF) and central venous pressure, right ventricular function, and lung fluid overload assessed by point-of-care ultrasound (POCUS) in hospitalized patients with acute heart failure (AHF).

**Methods:**

This was a prospective cohort study including AHF adult inpatients, conducted in Geneva University Hospitals from October 2019 to March 2020. The primary outcome was WRF, defined by an increase in creatinine of ≥1.5 times from baseline value or an increase of ≥0.3 mg/dL between admission and day 4 to 6. Expert ultrasonographers used POCUS to examine lungs, inferior vena cava during spontaneous expiration (IVCe), and tricuspid annular plane systolic excursion (TAPSE) at admission.

**Results:**

A total of 43 patients were included in the study. A total of 8 patients (19%) developed WRF during the study period (between October 8, 2019 and March 16, 2020), of whom 4 were in the higher quartile of lung fluid overload, 2 had TAPSE <14 mm, and 4 had IVCe ≥ 21 mm. In uni- and multi-variate logistic regression model, neither admission IVCe nor TAPSE was associated with WRF. However, lung congestion, as assessed by the number of B-lines, was significantly associated with WRF (odds ratio [OR] per quartile = 2.47, 95% confidence interval [CI] = 1.01 to 5.86, *P* = .04). This result remained statistically significant after adjustment for daily diuretic dose in mg/kg (OR = 2.98, 95% CI = 1.11 to 8.00, *P* = .03).

**Conclusion:**

This study showed that lung congestion as assessed by POCUS was associated with WRF in AHF patients, whereas IVCe and TAPSE were not. Due to the small number of participants, our results need to be prospectively validated in future adequately powered clinical trials.

## Abrégé

**Introduction:**

L’objectif de cette étude était d’évaluer l’association entre l’aggravation de la fonction rénale (AFR) et la pression veineuse centrale, la fonction ventriculaire droite et la surcharge liquidienne pulmonaire évaluées par échographie au point d’intervention (ÉPI) chez les patients hospitalisés pour insuffisance cardiaque aiguë (ICA).

**Méthodologie:**

Il s’agit d’une étude de cohorte prospective menée aux hôpitaux universitaires de Genève entre octobre 2019 et mars 2020 auprès de patients adultes hospitalisés pour ICA. Le principal critère d’évaluation était l’AFR, définie par une augmentation de la créatinine d’au moins 1,5 fois par rapport à la valeur initiale ou une augmentation d’au moins 0,3 mg/dl entre l’admission et les jours 4 à 6. Des experts en échographie ont utilisé l’ÉPI pour examiner les poumons, la veine cave inférieure pendant l’expiration spontanée (VCIe) et l’excursion systolique du plan de l’anneau tricuspide (TAPSE) à l’admission des patients.

**Résultats:**

L’étude a inclus 43 patients. Huit patients (19 %) ont développé une AFR au cours de la période de l’étude (entre le 8 octobre 2019 et le 16 mars 2020); de ceux-ci, quatre se trouvaient dans le quartile supérieur de surcharge liquidienne aux poumons, deux présentaient un TAPSE inférieur à 14 mm et quatre présentaient une VCIe d’au moins 21 mm. Dans les modèles de régression logistique univariée et multivariée, ni la VCIe ni le TAPSE mesurés à l’admission n’ont été associés à l’AFR. La congestion pulmonaire, évaluée par le nombre de lignes B, a par ailleurs été associée de façon significative à l’AFR (RC par quartile: 2,47; IC à 95 %: 1,01 à 5,86; *p* =,004). Ce résultat est demeuré statistiquement significatif après ajustement pour la dose quotidienne de diurétiques exprimée en mg/kg (RC: 2,98; IC à 95 %: 1,11 à 8,00 *p* =,003).

**Conclusion:**

Cette étude a montré que la congestion pulmonaire évaluée par ÉPI était associée à l’AFR chez les patients atteints d’ICA, ce qui n’a pas été le cas pour la VCIe et le TAPSE. En raison du faible nombre de participants, nos résultats doivent être validés prospectivement dans de futurs essais cliniques d’une puissance statistique suffisante.

## Introduction

Worsening renal function (WRF) during episodes of acute heart failure (AHF), also referred to as “cardiorenal syndrome type 1,”^1^ is a common finding and an independent predictor of poor outcomes.^2-6^ Heart and kidney dysfunctions are strongly interrelated; WRF has been classically attributed to low cardiac output; however, kidney venous congestion seems to have an important role to play in WRF pathogenesis.^7-9^ Increased central venous pressure (CVP), either caused by fluid overload or right ventricular dysfunction, is transmitted backward to the renal veins, possibly leading to increased kidney interstitial pressure and impaired glomerular filtration.^10^ In fact, elevated CVP is associated with reduced renal function in a variety of cardiovascular diseases^8^ and right rather than left ventricular dysfunction was shown to be associated with chronic kidney disease progression.^11^ Activation of the neuro-humoral system and fluid retention may lead to WRF by diminishing medullary perfusion pressure (the so-called renal tamponade hypothesis).^12^ Clinical evaluation of CVP and fluid overload is challenging for clinicians.^13^ Indeed, gold standard for CVP assessment is direct measurement of pressure in the superior vena cava or right atrium with catheterization. This kind of invasive equipment is, however, unpractical for daily clinical assessment. In addition, it is recognized that considerable disagreement and inaccuracy exist in the clinical assessment of CVP through jugular vein visual examination.^14,15^ This may lead to insufficient diuretic treatment and residual congestion at discharge, which is associated with worse clinical outcomes.^16,17^ Point-of-care ultrasonography (POCUS) outperforms physical examination for both fluid overload, right ventricular dysfunction, and CVP assessment.^18-20^ The aim of the present study was to investigate the association between WRF and POCUS-estimated CVP, right ventricular function, and lung fluid overload in hospitalized patients with AHF.

## Methods

This study was a pre-specified supplementary analysis of a prospective cohort study including AHF adult inpatients, conducted in Geneva University Hospitals from October 2019 to March 2020. Patients were recruited from the general internal medicine and cardiology inpatient units. Patients were routinely screened each working day by a study nurse for a diagnosis of AHF. Participation in the study was proposed to eligible patients within 72 hours of admission in the ward, and informed consent was obtained. Patients sequentially underwent a structured clinical examination, POCUS, and blood analysis twice: within 72 hours from admission (T0) and 4 to 6 days later (T1). Ultrasonographers were masked to clinical data files, and clinicians were blinded to POCUS findings. Paper case report forms were subsequently entered in the database with data entry verified by an independent data service provider (Data Conversion Service SA, Geneva, Switzerland). Baseline patient characteristics obtained from patients or electronic medical records were collected and documented by the study nurse. More details about study methods and patient recruitment are available in our previous report.^21^ Acute heart failure was defined according to the European Society of Cardiology criteria by the presence of at least 1 sign or symptom and a value of N-terminal pro-B type natriuretic peptide (NT-proBNP) of ≥300 ng/L.^22^ Serum creatinine baseline level was defined as the lowest value available in the year preceding hospital admission in the absence of dialysis.

### Point-of-Care Ultrasonography

Bedside ultrasonography was performed by expert sonographers using a high-end device (Philips SparQ, Philips AG Health Systems, Zurich, Switzerland) and a phased array probe (frequencies: 4-10 MHz) with preset 60% of gain and 15 cm of depth. Tissue harmonics were switched off for lung examination, allowing a better visualization of B-lines as previously described;^23^ a cardiac preset was selected (starting gain 50%, depth 15 cm). Patients laid supine with 30 to 45° bed elevation for lung ultrasound (LUS) and 0 to 30° for inferior vena cava during spontaneous expiration (IVCe) and tricuspid annular plane systolic excursion (TAPSE) measure. The thorax was explored bilaterally following a previously described 28-point protocol.^24^ The sum of the higher number of B-lines visualized in each point on a frozen image was calculated for the estimation of lung fluid overload. Right ventricular function was assessed in apical 4-chamber view measuring the TAPSE, the M-mode cursor positioned at the lateral portion of the tricuspid annulus with a cut-off for right ventricular systolic dysfunction (RVD) of <14 mm.^25,26^ Due to the existence of different cut-off values in the literature, a sensitivity analysis was performed with a cut-off value of 17 mm. Finally, as surrogate of CVP, inferior vena cava (IVC) size was measured in the subcostal view during unforced expiration (IVCe), using M-mode with a cut-off for high CVP of ≥21 mm.^27^ Only admission POCUS measures (T0) were used for the purpose of this study.

### Study Variables

**Figure 1.**
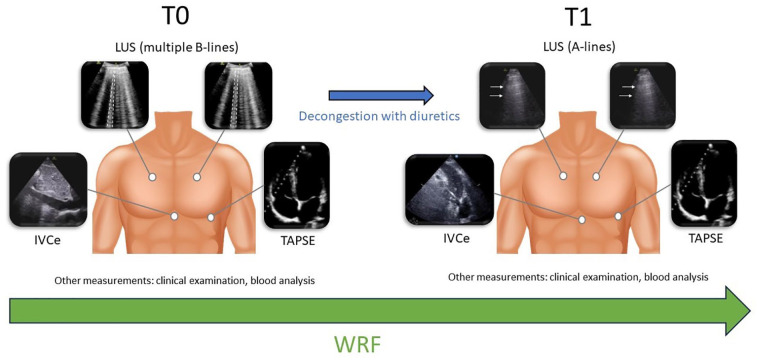
Representative image of each organ examined by ultrasound before and after decongestion. *Note.* T0, within 72 h from admission. T1, 4 to 6 days after T0. LUS = lung ultrasound. IVCe = inferior vena cava during spontaneous expiration. TAPSE = tricuspid annular plane systolic excursion. WRF = worsening renal function (primary outcome defined by an increase in creatinine of ≥1.5 times from baseline value or an increase of ≥0.3 mg/dL between admission and day 4 to 6).

The primary outcome was the development of WRF during the hospital stay. Worsening of renal function (WRF) was defined by an increase in creatinine of ≥1.5 from baseline value to T0 or T1 (worst value obtained) and/or an increase of ≥0.3 mg/dL (26.5 µmol/L) between T0 and T1. Predictor variables were the number of B-lines (in quartiles), TAPSE (below or above 14 mm), and IVCe size (below or above 21 mm) at T0. The total dose of diuretics from T0 to T1 was calculated retrospectively in daily normalized furosemide equivalents (mg/kg/day) by reviewing hospital electronic medical records. Results were adjusted for the normalized daily dose of diuretics. Figure 1 shows a representative image of each organ examined by ultrasound before and after decongestion.

### Statistical Analysis

Baseline characteristics of participants are presented as mean (standard deviation) or number (percentage), as appropriate.

To compare the incidence of the primary outcome across the B-line quartiles, TAPSE groups, or IVCe size groups, the chi-square or chi-square for trend was used, as appropriate. In addition, the association between WRF and POCUS-estimated lung fluid congestion (ie, B-lines quartiles), RVD (as estimated by TAPSE), or CVP (as estimated by IVCe) was assessed with univariate binary logistic regression. Results were adjusted in a multivariate model including the mean normalized daily dose of diuretics administered between T0 and T1 (mg/days/kg of intravenous [IV] furosemide equivalents). The small number of events prevented us from adjusting results for additional variables. P-values < .05 were considered statistically significant. SPSS software (version 27.0, SPSS Inc, Chicago, Illinois) was used for all statistical analyses.

## Results

Between October 8, 2019 and March 16, 2020, 43 patients were included in the study (mean age of 75 years; 23% of women, mean left ventricular ejection fraction of 43%; 28% with chronic kidney disease, defined as an estimated glomerular filtration rate < 60 ml/min/1.73 m^2^). Mean baseline creatinine was 101 ± 52 µmol/L; mean creatinine was 126 ± 63 µmol/L at T0 and 127 ± 70 µmol/L at T1. On average, patients received 1.1 ± 1.0 mg/kg/day of intravenous furosemide equivalents. Point-of-care ultrasonography was performed by 3 expert ultrasonographers within 1 day (interquartile range [IQR] = 1-3 days) after admission to the ward. Participants had an average total sum of 21.9 ± 14.7 B-lines on admission LUS. Significant pulmonary congestion was detected in 91% of study individuals on admission, as defined by the presence of ≥6 B-lines;^28^ one fifth (19.5%; 8/41) had abnormal value of TAPSE, and 58.5% (24/41) had enlarged IVCe. In 2 patients, an accurate value of TAPSE or IVCe could not be obtained for technical reasons. One patient who presented with WRF did not have an IVCe value on arrival. Key baseline characteristics are reported in Table 1; complete data are available in our previous report for this study.^21^ The NT-proBNP level was considerably higher in Quartile 4 than in the other groups (Table 1).

**Table 1.** Key Baseline Characteristics of the Study Population According to Admission B-Lines Quartiles, TAPSE, and IVCe Size.

	All patients	LUS quartiles, no. of B-lines (N = 43)				TAPSE, mm (N = 41)		IVCe, mm (N = 41)	
Characteristics	N = 43	Q1, 1-11 (N = 11)	Q2, 12-19 (N = 11)	Q3, 19-27 (N = 11)	Q4, >27 (N = 10)	<14 (N = 8)	≥14 (N = 33)	<21 (N = 17)	≥21 (N = 24)
Demographics									
Men, no. (%)	33 (76)	10 (90.9)	7 (63.6)	8 (72.7)	8 (80)	8 (100)	24 (72.7)	8 (47.1)	23 (95.8)
Age, mean (SD)	75 (11)	71 (12)	74 (11)	77 (10)	76 (14)	71 (12)	75 (12)	76 (10)	72 (12)
Comorbidities									
Diabetes, no. (%)	18 (42)	7 (63.6)	2 (18.2)	5 (45.5)	4 (40)	3 (37.5)	14 (42.4)	7 (41.2)	10 (41.7)
Hypertension, no. (%)	31 (72)	10 (90.9)	7 (63.6)	7 (63.6)	7 (70)	7 (87.5)	22 (66.7)	12 (70.6)	17 (70.8)
Prior heart failure, no. (%)	23 (54)	7 (63.6)	5 (45.5)	5 (45.5)	6 (60)	4 (50)	18 (54.5)	10 (58.8)	11 (45.8)
CKD, no. (%)	12 (28)	3 (27.3)	1 (9.1)	5 (45.5)	3 (30)	1 (12.5)	11 (33.3)	6 (35.3)	6 (25)
Medications									
Renin-angiotensin inhibitor, no. (%)	25 (58)	9 (81.8)	5 (45.5)	6 (54.5)	5 (50)	4 (50)	20 (60.6)	9 (52.9)	14 (58.3)
Beta-blocker, no. (%)	20 (46.5)	8 (72.7)	6 (54.5)	4 (36.4)	2 (20)	3 (37.5)	15 (45.5)	7 (41.2)	12 (50)
Diuretic dose in furosemide equivalent, mg/kg/d, (SD)	1.1 (1)	1.1 (0.9)	1.3 (1.6)	1 (0.9)	1 (0.7)	1.6 (2)	1 (0.7)	0.9 (0.6)	1.2 (1.3)
Laboratory tests									
Serum creatinine (µmol/L), mean (SD)	101 (52)	97 (31)	89 (25)	95 (38)	125 (90)	86 (16)	105 (58)	106 (76)	96 (28)
NT-proBNP (ng/L), mean (SD)	6742 (7076)	5075 (4507)	3616 (3151)	4294 (4605)	14707 (9127)	5345 (2791)	7410 (7823)	9238 (9932)	4772 (2918)

*Note.* Percentages are reported within each quartile or group. LUS = lung ultrasonography; TAPSE = tricuspid annular plane systolic excursion; IVCe = inferior vena cava during unforced expiration; CKD = chronic kidney disease; NT-proBNP = N-terminal pro-B type natriuretic peptide.

We assessed body weight and NT-proBNP values at T0 and T1. Weight dropped by a median of −1.2 kg (IQR = −3.3 to −0.7) and NT-proBNP by a median of −1220 ng/L (IQR = −4146 to −56).

**Figure 2.**
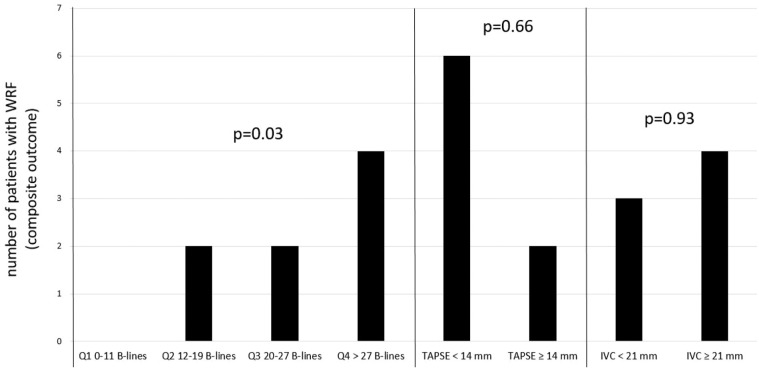
Association between B-line quartiles, TAPSE, or IVCe size and development of worsening of renal failure during hospital stay. *Note. P*-values for trend are provided. For binary variables, the chi-square *P*-value is depicted. WRF = worsening of renal function; TAPSE = tricuspid annular plane systolic excursion; IVCe = inferior vena cava during spontaneous expiration, Q1 = quartile 1; Q2 = quartile 2; Q3 = quartile 3; Q4 = quartile 4.

A total of 8 patients (19%) developed WRF during the study period, of whom 4 were in the higher quartile of lung fluid overload, 2 had TAPSE <14 mm, and 4 IVCe ≥ 2.1. The incidence of the primary outcome was higher in patients in the highest quartile for the number of B-lines (p-value for trend 0.03, Figure 2). In a univariate logistic regression model, neither IVCe nor TAPSE were associated with WRF (Figure 2 and Table 2). Results were similar when a TAPSE cut-off of <17 mm was used. More significant lung fluid overload, as estimated by the number of B-lines, however, significantly correlated with WRF as shown in Figure 2 and Table 2 (odds ratio (OR) = 2.47, 95% confidence interval (CI) = 1.01 to 5.86, *P* = .04). This result remained statistically significant after adjustment for normalized daily diuretic dose (OR = 2.98, 95% CI = 1.11 to 8.00, *P* = .03).

**Table 2.** Univariate and Multivariate Association Between B-Line Quartiles, TAPSE, or IVCe Size and Development of Worsening of Renal Function.

	Patients, no (%)	Unadjusted OR (95% CI)	*P*	Adjusted OR (95% CI)^a^	*P*
B-lines					
Q1	11/43 (25.6) ref				
Q2	11/43 (25.6)	2.47 (1.01-5.86)^b^	0.04	2.98 (1.11-8.00)^b^	.03
Q3	11/43 (25.6)				
Q4	10/43 (23.3)				
TAPSE					
<14 mm	8/41 (19.5)	1.50 (0.24-9.34)	0.66	0.97 (0.12-7.99)	.98
≥14 mm	33/41 (80.5) ref				
IVCe					
<21 mm	17/41 (41.5) ref				
≥21 mm	24/41 (58.5)	0.93 (0.18-4.84)	0.94	0.71 (0.12-4.10)	.70

a ORs are adjusted for daily normalized diuretic dose.

b For each incremental quartile.

We also examined change in B-lines in patients who developed WRF or not between T0 and T1. Patients without WRF had an average change in B-lines of −7.4 ± 2.2, while patients with WRF had an average change in B-lines of −13.5 ± 6.6 (difference between the 2 groups of 6.1, 95% CI = −5.1 to 17.3, *P* = .28). Similarly, there was no statistically significant difference in change in body weight or NT-proBNP between T0 and T1 in patients with and without WRF (*P* of 0.96 and 0.14, respectively).

## Discussion

In this prospective cohort study including 43 patients hospitalized for AHF, pulmonary fluid overload on admission evaluated by LUS significantly correlated with WRF, whereas echographic markers of RVD (TAPSE < 14 mm) and elevated CVP (IVC diameter ≥ 21 mm) did not.

Our main objective was to determine whether significant congestion, as assessed by LUS, could predict WRF during hospitalization. This association had been shown in the past in patients in the intensive care unit (ICU)^7^ using CVP measures, but it had never been shown with LUS. It has to be highlighted that patients with more severe congestion at presentation probably represent a sicker patient group, and this is explaining the identified association between the number of B-lines and incidence of WRF during the hospital stay.

Our sample size is inadequate to demonstrate whether changes in volume status during hospitalization, ie, better decongestion, are associated with changes in renal function or not. Although we did not find any significant difference in the change in the number of B-lines, weight, and NT-proBNP levels between T0 and T1 in patients with and without WRF, the small number of patients in our cohort precludes any definite conclusions with respect to the effect of decongestion on renal endpoints.

The LUS provides a semiquantitative assessment of pulmonary congestion, identifying extravascular lung water as B-lines.^29-33^ B-lines are significantly correlated with established parameters of AHF.^34^ Moreover, pulmonary congestion assessed by LUS is associated with a worse prognosis.^35^ Interestingly, in a recent randomized trial, a decongestive strategy mainly driven by IVC measurement did not impact short-term hospital readmission,^36^ whereas an LUS-guided decongestive therapy allowed a significant reduction in hospitalization in chronic HF patients with reduced ejection fraction.^37^ Moreover, during follow-up, lower rates of WRF were observed in patients allocated to the LUS-group, in comparison to those for whom decongestion rely on physical examination suggesting a role of subclinical fluid overload in cardiorenal syndrome pathogenesis. Remarkably, in a recent cohort of 39 inpatients with acute renal injury, a third of clinically euvolemic and a half of hypovolemic patients had moderate to severe lung fluid overload (ie, >15 B-lines).^38^ Thus, LUS in patients suffering from HF seems to outperform clinical examination in fluid overload assessment and decongestion guidance and might be useful to predict and prevent WRF secondary to acute cardiorenal syndrome.

A retrospective study by Mavrakanas et al^11^ demonstrated a significant, independent association between right ventricular diameter or function and chronic kidney disease (CKD) progression. The present study failed to find this association in patients with WRF in the context of AHF. This could be explained by inadequate power due to the small sample of our study or different pathophysiological mechanisms underlying acute and chronic kidney disease related to HF. Furthermore, there is a controversy about the normal value of TAPSE. A value below 14 mm is associated with a poor prognosis in patients with chronic heart failure, but even a value below 17 mm indicates RVD.^27,39^ In a post-hoc analysis using a TAPSE cut-off of <17 mm, however, there was still no significant association (results not shown).

Many physicians rely on IVC measurement to assess the volume status of patient. However, IVC measurement is associated with subjective variability^40^ and cannot be considered as a reliable standalone tool to assess CVP.^41^ Recently, Bhardwaj et al^42^ evaluated a modified VEXUS protocol (combination of IVC diameter, hepatic venous flow, and portal vein pulsatility index) in predicting acute kidney injury (AKI) in patients with cardiorenal syndrome. They found a strong correlation between grades of VEXUS and the stages of AKI. Renal venous ultrasound could also be an interesting tool.^43^ A recent study also described POCUS assessment of the jugular venous pressure and concluded that it is feasible, reproducible, and accurately predictive of elevated CVP in patients undergoing right heart catheterization.^44^

Another important point that needs to be highlighted is the clinical significance of WRF. It has been shown in the DOSE trial that patients with WRF actually had better clinical outcomes, compared with patients with improved renal function during hospitalization, which seems counterintuitive but could be possibly due to better decongestion in patients with WRF. Therefore, identification of patients at risk for AKI with B-line assessment should not preclude volume status optimization with adequate doses of diuretics, as small changes in creatinine levels during hospitalization may not represent AKI and are not related to hard clinical endpoints.^45,46^

This study has some limitations. First, the number of participants was modest due to recruitment interruption during the COVID-19 pandemic. The small number of events only allowed us to adjust results for daily normalized diuretic dose, precluding us to consider other potential confounders. Second, the 28-point LUS protocol used for this study has been largely validated, but it does not take into account the presence of pleural effusions, which is a very common marker of fluid overload. Other possible causes of WRF, such as postrenal injury, were not systematically investigated, and this may have influenced results; however, it is expected to lower the association more than strengthen it. Finally, non-cardiogenic pulmonary edema cannot be formally ruled out with LUS, and the higher number of B-lines in patients with WRF could potentially represent other causes not directly related to congestion.

To our knowledge, no study has yet shown a correlation between signs of congestion on LUS and WRF in the context of AHF, which is a strength of this study. However, our results remain hypothesis generating and will have to be prospectively validated in a future adequately powered clinical trial.
